# Supplementary material for: Complex‐centric proteome profiling by SEC‐SWATH‐MS
Source: Mol Syst Biol. 2019 Jan 14;15(1):e8438. doi: 10.15252/msb.20188438 (PMC6346213; doi:10.15252/msb.20188438)
Supplement: Supplementary file 8 — Dataset EV7 [file MSB-15-e8438-s008.zip › feature_plots_string/P11021.pdf]

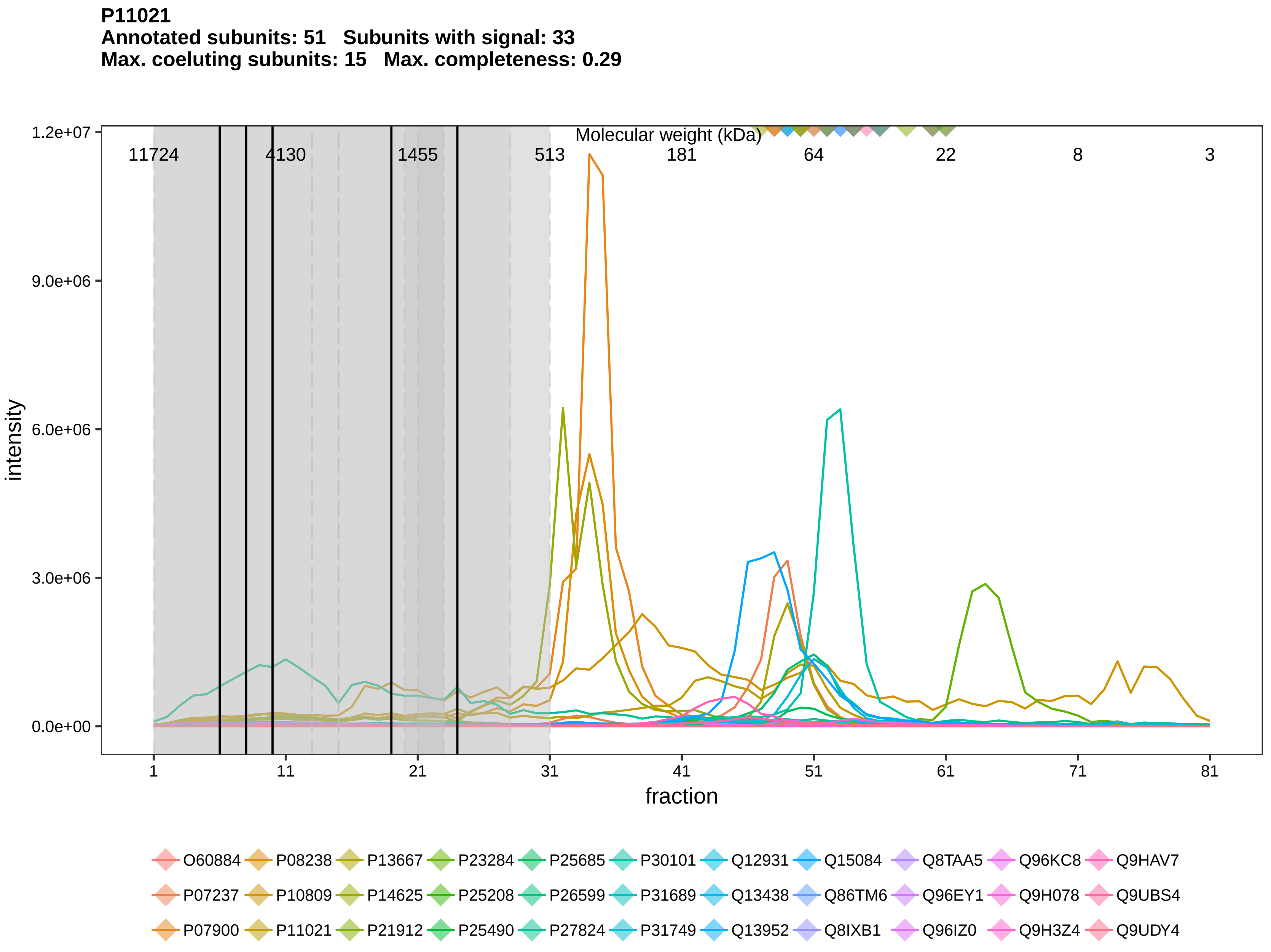

P11021
Annotated subunits: 51 Subunits with signal: 33
Max. coeluting subunits: 15 Max. completeness: 0.29
Molecular weight (kDa)
11724
4130
1455
513
181
64
22
8
3
intensity
1.2e+07
9.0e+06
6.0e+06
3.0e+06
0.0e+00
fraction
1
11
21
31
41
51
61
71
81
O60884
P08238
P13667
P23284
P25685
P30101
Q12931
Q15084
Q8TAA5
Q96KC8
Q9HAV7
P07237
P10809
P14625
P25208
P26599
P31689
Q13438
Q86TM6
Q96EY1
Q9H078
Q9UBS4
P07900
P11021
P21912
P25490
P27824
P31749
Q13952
Q8IXB1
Q96IZ0
Q9H3Z4
Q9UDY4
